# Survey of small intestinal and systemic immune responses following murine *Arcobacter butzleri* infection

**DOI:** 10.1186/s13099-015-0075-z

**Published:** 2015-10-19

**Authors:** Markus M. Heimesaat, Gül Karadas, Marie Alutis, André Fischer, Anja A. Kühl, Angele Breithaupt, Ulf B. Göbel, Thomas Alter, Stefan Bereswill, Greta Gölz

**Affiliations:** Department of Microbiology and Hygiene, Charité-University Medicine Berlin, Berlin, Germany; Institute of Food Hygiene, Freie Universität Berlin, Königsweg 69, 14163 Berlin, Germany; Department of Medicine I for Gastroenterology, Infectious Disease and Rheumatology/Research Center ImmunoSciences (RCIS), Charité-University Medicine Berlin, Berlin, Germany; Institute of Veterinary Pathology, Freie Universität Berlin, Berlin, Germany

**Keywords:** *Arcobacter butzleri*, Strain differences, Pro-inflammatory immune responses, Extra-intestinal sequelae, Systemic immune responses, Small intestine, Spleen, Apoptosis, Regenerating cells, Innate and adaptive immunity

## Abstract

**Background:**

*Arcobacter* (*A.*) *butzleri* has been described as causative agent for sporadic cases of human gastroenteritis with abdominal pain and acute or prolonged watery diarrhea. In vitro studies revealed distinct adhesive, invasive and cytotoxic properties of *A. butzleri*. Information about the underlying immunopathological mechanisms of infection in vivo, however, are scarce. The aim of this study was to investigate the immunopathological properties of two different *A.butzleri* strains in a well-established murine infection model.

**Results:**

Gnotobiotic IL-10^−/−^ mice, in which the intestinal microbiota was depleted by broad-spectrum antibiotic treatment, were perorally infected with two different *A. butzleri* strains isolated from a diseased patient (CCUG 30485) or fresh chicken meat (C1), respectively. Eventhough bacteria of either strain could stably colonize the intestinal tract at day 6 and day 16 postinfection (p.i.), mice did not exert infection induced symptoms such as diarrhea or wasting. In small intestines of infected mice, however, increased numbers of apoptotic cells could be detected at day 16, but not day 6 following infection with either strain. A strain-dependent influx of distinct immune cell populations such as T and B cells as well as of regulatory T cells could be observed upon *A. butzleri* infection which was accompanied by increased small intestinal concentrations of pro-inflammatory cytokines such as TNF, IFN-γ, MCP-1 and IL-6. Remarkably, inflammatory responses following *A. butzleri* infection were not restricted to the intestinal tract, given that the CCUG 30485 strain induced systemic immune responses as indicated by increased IFN-γ concentrations in spleens at day 6, but not day 16 following infection.

**Conclusion:**

Upon peroral infection *A. butzleri* stably colonized the intestinal tract of gnotobiotic IL-10^−/−^ mice. The dynamics of distinct local and systemic inflammatory responses could be observed in a strain-dependent fashion pointing towards an immunopathogenic potential of *A. butzleri* in vivo. These results indicate that gnotobiotic IL-10^−/−^ mice are well suited to further investigate the molecular mechanisms underlying arcobacteriosis in vivo.

## Background

*Arcobacter* and *Campylobacter* share taxonomic relationship given that the genus *Arcobacter* belongs to the family of *Campylobacteraceae* [1]. So far, 18 distinct *Arcobacter* species have been described, isolated from a plethora of environments and hosts [1]. Whereas the motile, spiral-shaped and gram-negative *Arcobacter* spp. are mostly reported as gastrointestinal commensals in animals, the International Commission on Microbiological Specifications for Foods has rated *Arcobacter* (*A*.) *butzleri* and *A. cryaerophilus* as serious hazards for human health [2]. Robust epidemiological data regarding prevalence and incidence of *Arcobacter* associated human diseases are rather scarce, since in the vast majority of cases *Arcobacter* spp. are not detected by routine diagnostic measures applied in conventional microbiology laboratories [1]. Retrospective studies, however, revealed that *Arcobacter* spp. are the fourth most common *Campylobacterales* species recovered from patients suffering from diarrhea [3–7]. Consumption of contaminated food or water has been considered the most likely mode of transmission leading to *Arcobacter* induced disease outbreaks [6, 8]. Arcobacteriosis is characterized by symptoms of acute gastroenteritis including abdominal pain, acute diarrhea or prolonged watery diarrhea for up to several weeks [5–7]. So far, only very limited information is available about the underlying immunopathogenic mechanisms and responsible virulence genes of *Arcobacter* infection. Ten putative virulence genes, namely *cadF*, *mviN*, *pldA*, *tlyA*, cj1349, *hecB*, *irgA*, *hecA*, *ciaB* and *iroE* have been detected within the genomic sequence of *A.* *butzleri* strain RM 4018 [9]. These virulence factors have been shown to contribute to adhesion (CadF, HecA, Cj1349), invasion (CiaB), lysis of erythrocytes (HecB, TlyA, PldA), iron acquisition and maintenance of infection (IrgA, IroE) and peptidoglycan biosynthesis (MviN) in other bacteria [10–18]. Nevertheless, it is still unknown whether these putative virulence factors exert similar functions in *Arcobacter* and whether further virulence genes exist within these species. Phenotypic assays revealed that *A. butzleri* is able to adhere to and invade into several cell lines. However, there is no correlation between distinct virulence gene patterns of *A. butzleri* isolates and adhesive and invasive properties in vitro [19, 20]. Also cytotoxic effects of several *A. butzleri* strains have been observed in vitro, but no corresponding toxin has been identified yet [4, 21–25].

Data regarding the immunopathological mechanisms underlying *A. butzleri* infection and the corresponding host responses are scarce and conflicting. In vivo studies revealed discrepant results so far, depending on the animal species, the breed and on the respective *A. butzleri* strain. For instance, *A. butzleri* was unable to infect conventional chicken and induce disease, whereas certain turkey strains could be colonized at different loads and displayed variable mortality rates [26]. Furthermore, in neonatal piglets *A. butzleri* displayed rather invasive properties and could also be isolated from extra-intestinal compartments including kidney, liver and brain [27]. Whereas neonatal albino rats presented with self-limiting diarrhea and small intestinal as well as hepatic necrosis, adult rats exerted watery diarrhea and disturbed serum electrolyte balance in a pathogen-load-dependent manner [28, 29].

Murine infection experiments with enteric pathogens such as *Campylobacter jejuni* are hampered by the physiological colonization resistance exerted by conventionally colonized mice due to their distinct host- and age-specific microbiota composition [30]. We have previously reported that following depletion of the murine intestinal microbiota in IL-10^−/−^ mice by broad-spectrum antibiotic treatment, colonization resistance could be overcome subsequently facilitating infection induced immunpathological sequelae such as acute enterocolitis which is a key feature of human campylobacteriosis [31–33]. Given that *C. jejuni* and *A. butzleri* share taxonomic relationships we used the gnotobiotic IL-10^−/−^ mouse model to determine the pathogenic potential of *A. butzleri* and to investigate its host-interactions in the murine intestinal tract. Hereby, we focussed on the small intestines given that certain bacterial species use the ileal membranous epithelial cells (M cells) for invasion and subsequent induction of inflammation [34]. To accomplish this, gnotobiotic IL-10^−/−^ mice were perorally infected with one of two different *A. butzleri* strains [19, 35]. Then, colonization properties alongside the intestinal tract, translocation of viable bacteria to distant extra-intestinal compartments, induction of histopathological changes including apoptosis and, finally, local (i.e. small intestinal) as well as systemic pro-inflammatory responses were analyzed.

## Results

### Abundance of *A. butzleri* in the intestinal tract following peroral infection of gnotobiotic IL-10^−/−^ mice

**Fig. 1:**
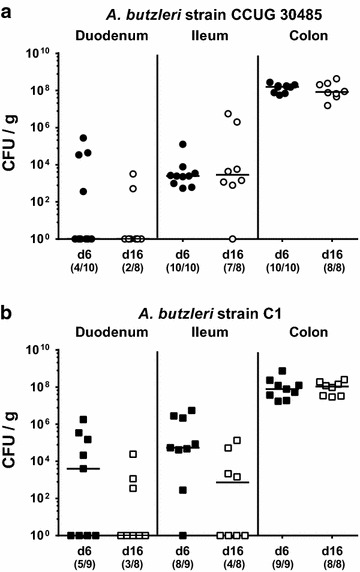
Colonization of *Arcobacter butzleri* alongside the murine intestinal tract following peroral infection. Gnotobiotic IL-10^−/−^ mice were generated by antibiotic treatment and orally infected either with **a** *A. butzleri* strain CCUG 30485 (*circles*) or **b** strain C1 (*squares*). *A. butzleri* loads were determined in luminal samples of duodenum, ileum and colon at day 6 p.i. (*filled symbols*) and day 16 p.i. (*open symbols*) as colony forming units (CFU) per gram sample. Medians (*black bars*) and numbers of mice harboring the pathogen out of the total number of analyzed animals (in *parentheses*) are indicated. Data shown were pooled from three independent experiments

The induction of small intestinal and systemic immune responses were investigated in mice infected with the *A. butzleri* strains CCUG 30485 and C1. Both *A. butzleri* strains encode for all ten putative virulence genes and displayed adhesive and invasive phenotypes in human epithelial cell culture models [19]. The reference strain CCUG 30485 was initially isolated from a diseased patient [35], whereas the C1 strain was derived from fresh chicken meat [19]. In order to overcome physiological colonization resistance of mice harboring a conventional microbiota preventing from infection with pathogens, we generated gnotobiotic mice by depleting the intestinal microbiota following broad-spectrum antibiotic treatment [30]. Previous *C. jejuni* infection studies revealed that the murine gnotobiotic IL-10^−/−^ model is well suited not only to study pathogenic colonization properties, but also infection-induced immunopathological responses mimicking key features of human disease [31–33]. Furthermore, given the taxonomic relationship of *Arcobacter* and *Campylobacter*, we applied the gnotobotic IL-10^−/−^ model to unravel colonization and immunopathological properties of *A. butzleri* in the present study. Given that *C. jejuni* induced non-self-limiting enterocolitis in gnotobiotic IL-10^−/−^ mice within 6 days p.i. [31–33], we investigated potential immunopathological sequelae of *A. butzleri* infection at day 6 p.i. and, additionally, to a later time point, namely day 16 p.i. Six and 16 days following peroral infection with 10^9^ viable *A. butzleri* the respective isolates could be cultured from the small and large intestinal lumen with highest loads of up to 10^8^ colony forming units (CFU) per g in the colon and approximately 10^4^ CFU per g in the ileum of infected gnotobiotic IL-10^−/−^ mice (Fig. 1). Colonization densities of either strain did not differ between day 6 and day 16 p.i. in the respective intestinal compartment. Hence, *A. butzleri* of either strain is able to stably establish alongside the intestinal tract in the course of infection. Interestingly, mice did not exhibit any clinical signs of enteric disease such as wasting, diarrhea or occurence of blood in stool.

### Macroscopic and microscopic aspects of small intestinal inflammatory sequelae in *A. butzleri* infected gnotobiotic IL-10^−/−^ mice

Given that intestinal inflammation is accompanied by significant shortening of the intestinal tract [31, 36], we determined small intestinal lengths at days of necropsy. Neither at day 6 nor at day 16 following *A. butzleri* infection with either strain, shortening of the small intestines could be observed when compared to uninfected control mice (not shown), further supporting an absence of acute infection-induced macroscopic disease.

We next surveyed potential microscopic intestinal sequelae of infection in hematoxylin & eosin (H&E) stained small intestinal paraffin sections applying a standardized histopathological scoring system (see “Methods”). Histopathological scores did not differ between naive and infected mice irrespective of the applied strain and the time point (day 6 and 16 p.i.; not shown).

**Fig. 2:**
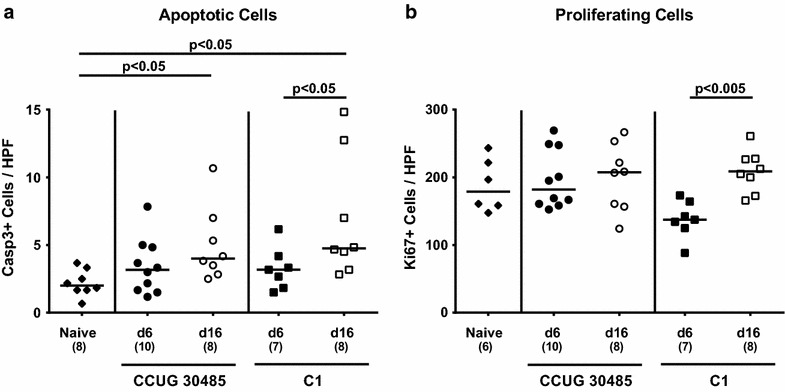
Kinetics of apoptotic and proliferating cells in ileal tissue following murine *A. butzleri* infection. Gnotobiotic IL-10^−/−^ mice were generated by antibiotic treatment and orally infected either with *A. butzleri* strain CCUG 30485 (*circles*) or strain C1 (*squares*). Uninfected gnotobiotic IL-10^−/−^ mice served as negative control (*black diamonds*). The average numbers of apoptotic (positive for caspase-3, Casp3; **a**) and proliferating cells (positive for Ki67; **b**) from at least six high power fields (HPF, ×400 magnification) per animal were determined microscopically in immunohistochemically stained ileal section at day 6 p.i. (*filled symbols*) and day 16 p.i. (*open symbols*). Numbers of analyzed animals are given in *parenthesis*. Medians (*black bars*) and level of significance (*p* value) determined by Mann–Whitney U test are indicated. Data shown were pooled from three independent experiments

We next assessed numbers of caspase-3+ cells within the small intestinal mucosa of infected mice given that apoptosis is a commonly used diagnostic marker in the histopathological evaluation and grading of intestinal disease [30] and a hallmark of *C. jejuni* induced enterocolitis in gnotobiotic IL-10^−/−^ mice [31]. At day 16, but not earlier at day 6 following infection with the CCUG 30485 or C1 strain, mice displayed higher apoptotic cells in the epithelial cells of the small intestinal mucosa as compared to naive control animals (p < 0.05; Fig. 2a). Since Ki67 comprises a nuclear protein necessary for cellular proliferation [37], we quantitatively determined proliferating cells in small intestinal paraffin sections in situ. Following C1 strain infection only, gnotobiotic IL-10^−/−^ mice displayed more Ki67+ small intestinal epithelial cells at day 16 as compared to day 6 p.i. (p < 0.005; Fig. 2b).

### Small intestinal influx of distinct immune cell populations in *A. butzleri* infected gnotobiotic IL-10^−/−^ mice

**Fig. 3:**
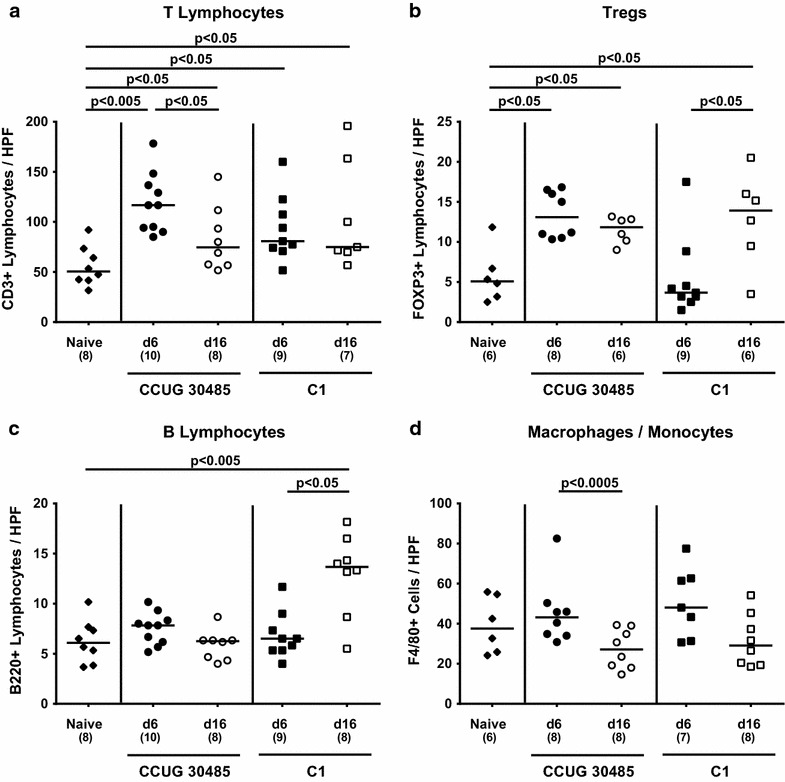
Kinetics of immune cell infiltration in ileal tissue following murine *A. butzleri* infection. Gnotobiotic IL-10^−/−^ mice were generated by antibiotic treatment and orally infected either with *A. butzleri* strain CCUG 30485 (*circles*) or strain C1 (*squares*). Uninfected gnotobiotic IL-10^−/−^ mice served as negative control (*black diamonds*). The average number of cells positive for **a** CD3 (T lymphocytes). **b** FOXP3 (regulatory T cells, Tregs). **c** B220 (B lymphocytes) and **d** F4/80 (macrophages and monocytes) from at least six high power fields (HPF, ×400 magnification) per animal were determined microscopically in immunohistochemically stained ileal sections derived from mice at day 6 p.i. (*filled symbols*) and day 16 p.i. (*open symbols*). Numbers of analyzed animals are given in *parentheses*. Medians (*black bars*) and significance levels as determined by the Mann–Whitney U test are indicated. Data shown were pooled from three independent experiments

Given that recruitment of innate and adaptive immune cells as well as of effector cells to sites of inflammation is a hallmark of enteric infection such as campylobacteriosis [30], we next quantitatively assessed distinct immune cell populations by in situ immunohistochemical staining of small intestinal paraffin sections. Irrespective of the *A. butzleri* strain, numbers of small intestinal CD3+ T lymphocytes increased 6 and 16 days following infection (p < 0.05–0.005; Fig. 3a). Remarkably, numbers of T cells as well as of FOXP3+ regulatory T cells (Tregs) increased by more than twofold as early as 6 days following CCUG 30485 strain infection (p < 0.005 and p < 0.05, respectively; Fig. 3a, b), whereas the T cells declined thereafter to a still elevated level (p < 0.05; Fig. 3a). Furthermore, numbers of small intestinal B220+ B lymphocytes increased rather late (i.e. until day 16 p.i.) during *A. butzleri* C1 strain, but not CCUG 30485 strain infection **(**p < 0.005; Fig. 3c). The influx of macrophages and monocytes into the small intestinal tract upon *A. butzleri* infection, however, was not enhanced as indicated by comparable ileal F4/80+ cell numbers in infected and uninfected mice at either time point (Fig. 3d).

### Small intestinal and systemic pro-inflammatory cytokine secretion in *A. butzleri* infected gnotobiotic IL-10^−/−^ mice

**Fig. 4:**
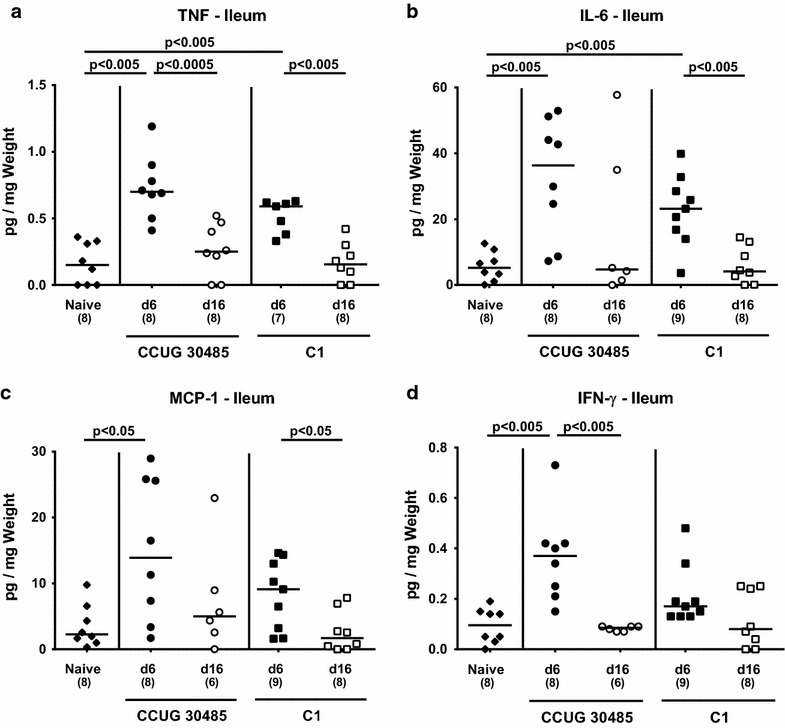
Kinetics of pro-inflammatory cytokine responses in the ileum following murine *A. butzleri* infection. Gnotobiotic IL-10^−/−^ mice were generated by antibiotic treatment and orally infected either with *A. butzleri* strain CCUG 30485 (*circles*) or strain C1 (*squares*). Uninfected gnotobiotic IL-10^−/−^ mice served as negative control (*black diamonds*). Concentrations (pg per mg ileal tissue) of **a** TNF, **b** IL-6, **c** MCP-1 and **d** IFN-γ were determined in supernatant of ex vivo ileal biopsies at day 6 p.i. (*filled symbols*) and day 16 p.i. (*open symbols*). Numbers of analyzed animals are given in *parentheses*. Medians (*black bars*) and significance levels as determined by the Mann–Whitney U test are indicated. Data shown were pooled from three independent experiments

**Fig. 5:**
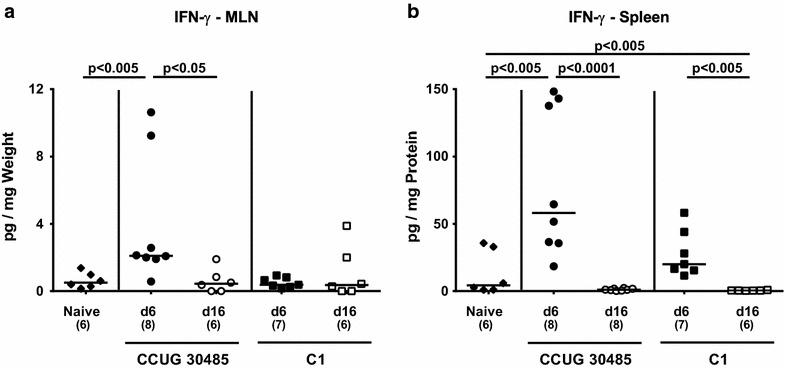
Kinetic of IFN-γ responses in ex vivo biopsies of mesenteric lymphnodes and spleens of *A. butzleri* infected mice. Gnotobiotic IL-10^−/−^ mice were generated by antibiotic treatment and orally infected either with *A. butzleri* strain CCUG 30485 (*circles*) or strain C1 (*squares*). Uninfected gnotobiotic IL-10^−/−^ mice served as negative control (*black diamonds*). Concentrations (pg per mg of total protein) of IFN-γ were determined in supernatant of ex vivo biopsies derived from **a** mesenteric lymphnodes (MLNs) and **b** spleens at day 6 p.i. (*filled symbols*) and day 16 p.i. (*open symbols*). Numbers of analyzed animals are given in *parentheses*. Medians (*black bars*) and significance levels as determined by the Mann–Whitney U test are indicated. Data shown were pooled from three independent experiments

We next determined the secretion of pro-inflammatory cytokines in supernatants of ex vivo biopsies derived from ileum, mesenteric lymphnodes (MLNs) and spleen. At day 6, but not day 16 following *A. butzleri* infection with either strain, higher TNF and IL-6 levels could be detected in the small intestine as compared to naive mice (p < 0.005; Fig. 4a, b). Ileal MCP-1 concentrations increased upon CCUG 30485 strain infection until day 6 (p < 0.05; Fig. 4c), and were higher at 6 days as compared to 16 days following C1 strain infection (p < 0.05; Fig. 4c). Furthermore, IFN-γ concentrations were elevated in small intestines as well as in MLNs as early as 6 days following CCUG 30485 strain infection (p < 0.005; Fig. 4d; p < 0.005; Fig. 5), but declined back to naive levels until day 16 p.i. (p < 0.005; Fig. 4d; p < 0.05; Fig. 5a). Strikingly, this held also true for IFN-γ levels in spleens that increased 6 days, but not 16 days following CCUG 30485 strain infection (p < 0.005; Fig. 5b), indicating that *A.butzleri* infection resulted not only in local but also systemic immune responses in a strain-dependent fashion.

Taken together, despite absence of clinical symptoms of disease and stable colonization alongside the intestinal tract, peroral *A. butzleri* infection led to small intestinal apoptosis, induced influx of T and B cells as well as of Tregs to sites of infection and resulted in intestinal and systemic pro-inflammatory cytokine responses. Taken together, these findings underline a distinct immunopathogenic potential of *A. butzleri* in vivo.

## Discussion

The pathogenic relevance of *Arcobacter* infections in humans is under current debate. Overall, solid epidemiological data are rather scarce. One of the reasons might be that species identification by the currently established routine measures in conventional microbiology laboratories is rather challenging. It is therefore highly likely that the reported cases—mostly sporadic outbreaks due to contaminated food or water—are highly under-representing the real prevalence [1]. Eventhough *Campylobacter* and *Arcobacter* are taxonomically related and, furthermore, in the meantime *C. jejuni* has established as the most commonly reported bacterial etiological agent of diarrhea in developed countries outcompeting *Salmonella* [38], the scientific community did not undertake ambitious efforts to unravel potencies of *Arcobacter* in inducing immunopathology in vivo. Results from the few studies to date, however, are rather conflicting and highly dependent on the animal species, the breed and/or on the respective *A. butzleri* strain under investigation [1]. To our knowledge, only one single *Arcobacter* infection study has been performed in mice so far demonstrating that following serial intraperitoneal passages in mice, the adherent capabilities of initially low-adherent *A. butzleri* strains were enhanced [39]. Previously, our group has established several murine *C. jejuni* infection models (reviewed in [40]). To assure proper pathogenic colonization of the murine gut, the microbiota needs to be modified to overcome the physiological colonization resistance [30]. Among these models, gnotobiotic IL-10^−/−^ mice, in which the intestinal microbiota was depleted by broad-spectrum antibiotic treatment, develop acute enterocolitis within 1 week following oral *C. jejuni* infection mimicking key features of severe human campylobacteriosis [31–33]. Given the close relationship between *Arcobacter* and *Campylobacter* we applied the gnotobiotic IL-10^−/−^ mouse model to unravel the immunopathological impact of two *A. butzleri* strains derived from two different hosts (namely human and chicken) and the respective bacterial-host-interactions in the present study. Following peroral infection either *A. butzleri* strain could stably colonize the intestinal tract with comparable loads. Whereas the colon harbored the highest *A. butzleri* densities, approximately four orders of magnitude lower bacterial loads were detected in the ileal lumen. It is of note that mice did not exert any overt infection induced symptoms such as diarrhea or wasting. This is somewhat surprising, however, given that the two *A. butzleri* strains have been shown to exert adhesive and invasive properties in vitro [19]. The absence of macroscopic signs of disease might also be attributable to a lower pathogenic potential as compared to *C. jejuni*. This might indicate that the observed symptoms induced by *A. butzleri* in our gnotobiotic IL-10 deficient mice mimic arcobacteriosis in humans, which is thought to cause milder disease symptoms as compared to human campylobacteriosis. However, infected mice developed increased small intestinal caspase-3+ apoptosis at day 16 p.i., and hence rather late in comparison to *C. jejuni* infection in the same murine model. However, this is in line with results from in vitro studies showing that *A. butzleri* induced apoptosis in intestinal epithelial cells [41]. Furthermore, in human macrophages *A. butzleri* infection resulted in a distinct pro-inflammatory response as indicated by highly upregulated expression levels of TNF, IL-6 and IL-12 (like in our in vivo study), whereas the anti-inflammatory cytokine IL-10 was moderately upregulated. This is further underlining that the murine gnotobiotic IL-10^−/−^ applied here is well suited to unravel pathogen-host-interactions. Despite subsequent activation of caspase-3, -7 and -8, macrophages survived *A. butzleri* infection without any signs of DNA damage pointing towards cellular counter-regulatory measures [42]. Given that in our study higher ileal apoptotic as well as Ki67+ proliferating cell numbers could be observed at day 16 as compared to day 6 following C1 strain infection, absence of macroscopic disease might have also been due to distinct counter-regulatory measures despite stable bacterial colonization. Despite the absence of macroscopic disease symptoms, *A. butzleri* induced a prominent influx of pro-inflammatory innate and adaptive immune cell populations such as T and B cells as well as Tregs into the small intestinal mucosa and lamina propria. In another in vivo study, *A. butzleri* could be cultured from the small intestines of Cesarean-derived neonatal piglets [27]. The strain exerted invasive properties, given that viable bacteria could be isolated from extra-intestinal organs including kidney, lung and brain. Also in our study, the inflammatory responses following peroral *A. butzleri* infection were not restricted to the intestinal tract, since IFN-γ levels were not only elevated in ilea and MLNs, but also in spleens of CCUG 30485 strain infected mice early in the course of disease. Notably, we were not able to isolate viable pathogens from extra-intestinal compartments including the spleen of infected mice by direct plating. Except for reddening of the ileal mucosa in single piglets, neither gross nor microscopic signs could be observed in the small intestinal tract of *A. butzleri* infected neonatal piglets [27]. In another in vivo study, albino rats presented with self-limiting diarrhea 5 days following peroral *A.* *butzleri* infection that was accompanied with leukocytic infiltrates in the intestinal lamina propria [43]. These results are well in line with our data given that increased numbers of ileal T and B cells, but also of regulatory T cells could be observed in the course of *A. butzleri* infection of gnotobiotic IL-10^−/−^ mice.

The rather heterogenous outcome of *A. butzleri* infection in vivo might be attributed to a plethora of distinct contextual factors such as host-related differences including animal species, age, immune status and microbiota composition on the host side and bacterial strain-dependent factors [1]. In our study, distinct strain-dependent differences in pathogen-host-interaction could be observed in infected gnotobiotic IL-10^−/−^ mice. Given that *Arcobacter* strains express variable lipopolysaccharide (LPS) or lipooligosacchardie (LOS) structures, this might determine whether the respective strains rather act as commensals or as pathogens in vivo. Previously, we have demonstrated that *C. jejuni* infection is mediated by TLR-4-dependent signalling of bacterial LOS [30, 31]. Accordingly, we are currently investigating potential TLR-4-dependent intestinal, extra-intestinal as well as systemic immunopathological features of arcobacteriosis in murine in vivo models.

## Conclusion

In conclusion, in perorally infected gnotobiotic IL-10^−/−^ mice *A. butzleri* induce small intestinal and systemic pro-inflammatory immune responses in a strain-dependent fashion. The immunopathogenic potential of *A. butzleri* in vivo indicates that the murine model applied here is well suited to further unravel the molecular mechanisms underlying pathogen-host-interactions during acrobacteriosis.

## Methods

### Ethics statement

All animal experiments were conducted according to the European Guidelines for animal welfare (2010/63/EU) with approval of the commission for animal experiments headed by the “Landesamt für Gesundheit und Soziales” (LaGeSo, Berlin, registration number G0184/12). Animal welfare was monitored twice daily by assessment of clinical conditions.

### Mice

IL-10^−/−^ mice (in C57BL/6j background, B6) were bred and maintained in the facilities of the “Forschungseinrichtungen für Experimentelle Medizin” (FEM, Charité-Universitätsmedizin, Berlin, Germany) under specific pathogen-free (SPF) conditions.

Gnotobiotic IL-10^−/−^ mice (with a virtually depleted gastrointestinal microbiota) were generated following broad-spectrum antibiotic treatment as described earlier [31, 36]. In brief, mice were transferred to sterile cages and treated by adding ampicillin/sulbactam (1 g/L; Pfizer, Berlin, Germany), vancomycin (500 mg/L; Hexal, Holzkirchen, Germany), ciprofloxacin (200 mg/L; Hexal), imipenem (250 mg/L; Fresenius Kabi, Graz, Austria), and metronidazole (1 g/L; Braun, Melsungen, Germany) to the drinking water ad libitum starting at 3 weeks of age immediately after weaning and continued for 3 months before the infection experiment [33]. Three days prior infection, the antibiotic cocktail was replaced by sterile tap water (ad libitum). Mice were continuously kept in a sterile environment (autoclaved food and tap water) and handled under strict antiseptic conditions.

### *Arcobacter butzleri* infection of mice

Gnotobiotic IL-10^−/−^ mice were perorally infected with approximately 10^9^ viable CFU of two different *Arcobacter butzleri* strains either (CCUG 30485 or C1 strain, respectively) by gavage in a total volume of 0.3 mL phosphate buffered saline (PBS) on two consecutive days (day 0 and day 1). Naive age- and sex-matched gnotobiotic IL-10^−/−^ mice served as uninfected controls.

The *A. butzleri* reference strain CCUG 30485 was initially isolated from a fecal sample derived from a diarrheal patient [35], whereas the C1 strain was isolated from fresh chicken meat [19]. Both *A. butzleri* strains were grown on Karmali-Agar (Oxoid, Wesel, Germany) for 2 days at 37 °C under microaerobic conditions using CampyGen gas packs (Oxoid).

### Clinical score

To assess clinical signs of *A. butzleri* infection on a daily basis, a standardized cumulative clinical score (maximum 12 points), addressing the occurrence of blood in feces (0: no blood; 2: microscopic detection of blood by the Guajac method using Haemoccult, Beckman Coulter/PCD, Krefeld, Germany; 4: overt blood visible), diarrhea (0: formed feces; 2: pasty feces; 4: liquid feces), and the clinical aspect (0: normal; 2: ruffled fur, less locomotion; 4: isolation, severely compromised locomotion, pre-final aspect) was used [31].

### Sampling procedures

Mice were sacrificed by isoflurane treatment (Abbott, Greifswald, Germany) on day 6 or day 16 p.i.. Tissue samples from MLNs, spleen, and ileum were removed under sterile conditions. Absolute small intestinal lengths were determined by measuring the distances from the transition of the stomach to the duodenum to the very distal terminal ileum by a ruler. Ileal ex vivo biopsies from each mouse were collected in parallel for immunohistochemical, microbiological, and immunological analyses. Immunohistopathological changes were determined in ileal samples immediately fixed in 5 % formalin and embedded in paraffin. Sections (5 μm) were stained with H&E or respective antibodies for in situ immunohistochemistry as described earlier [33].

### Histopathological grading of small intestinal lesions

To evaluate the severity of small intestinal histopathological lesions, an established scoring scheme [44] with minor modifications was applied. In detail, the composition of the immune cell infiltrates (0: none; 1: mononuclear cells; 2: mononuclear cell dominated, fewer neutrophils; 3: neutrophil dominated, fewer mononuclear cells), quantity of the immune cell infiltrates (0: none; 1: mild; 2: moderate; 3: severe), vertical extent of inflammation (0: none; 1: mucosa; 2: mucosa and submucosa; 3: transmural), and horizontal extent of inflammation (0: no; 1: focal; 2: multifocal; 3: multifocal-coalescent; 4: diffuse) were assessed. The cumulative histologic scoring ranged from 0 to 13 for ileal samples.

### Immunohistochemistry

In situ immunohistochemical analysis of ileal paraffin sections was performed as described previously [30, 31, 45–47]. Primary antibodies against cleaved caspase-3 (Asp175, Cell Signaling, Beverly, MA, USA, 1:200), Ki67 (TEC3, Dako, Denmark, 1:100), CD3 (#N1580, Dako, 1:10), FOXP3 (FJK-16 s, eBioscience, San Diego, CA, USA, 1:100), B220 (eBioscience, 1:200), and F4/80 (# 14-4801, clone BM8, eBioscience, 1:50), were used. For each animal, the average number of positively stained cells within at least six high power fields (HPF, 0.287 mm^2^, 400× magnification) were determined microscopically by a double-blinded investigator.

### Quantitative analysis of *Arcobacter butzleri*

Viable *A. butzleri* were detected in feces or at time of necropsy (day 6 or 16 p.i.) in luminal samples taken from the duodenum, ileum or colon, dissolved in sterile PBS and cultured in serial dilutions on Karmali- and Columbia-Agar supplemented with 5 % sheep blood (Oxoid) in parallel for 2 days at 37 °C under microaerobic conditions using CampyGen gas packs (Oxoid). To quantify bacterial translocation, MLNs and spleen (≈1 cm^2^) were homogenized in 1 mL sterile PBS, streaked onto Karmali-Agar and cultivated accordingly. The respective weights of fecal or tissue samples were determined by the difference of the sample weights before and after asservation. The detection limit of viable pathogens was ≈100 colony forming units (CFU) per g.

### Cytokine detection in culture supernatants of ex vivo biopsies taken from ileum, mesenteric lymphnodes and spleen

Ileal ex vivo biopsies were cut longitudinally and washed in PBS. Spleen, MLNs or strips of approximately 1 cm^2^ ileum were placed in 24-flat-bottom well culture plates (Nunc, Wiesbaden, Germany) containing 500 μL serum-free RPMI 1640 medium (Gibco, life technologies, Paisley, UK) supplemented with penicillin (100 U/mL) and streptomycin (100 µg/mL; PAA Laboratories). After 18 h at 37 °C, culture supernatants were tested for IFN-γ, TNF, MCP-1, IL-6 and IL-12p70 by the Mouse Inflammation Cytometric Bead Assay (CBA; BD Biosciences, San Jose, CA, USA) on a BD FACSCanto II flow cytometer (BD Biosciences). Nitric oxide (NO) was determined by Griess reaction as described earlier [36].

### Statistical analysis

Medians and levels of significance were determined using Mann–Whitney test (GraphPad Prism v5, La Jolla, CA, USA). Two-sided probability (*P*) values ≤0.05 were considered significant. Experiments were reproduced twice.
